# Gender role attitudes and well-being of German and refugee adolescents—same or different?

**DOI:** 10.1186/s12888-023-05100-4

**Published:** 2023-09-08

**Authors:** Hannah Nilles, Usama EL-Awad, Denny Kerkhoff, Johanna Braig, Pia Schmees, Yasemin Kilinc, Jana-Elisa Rueth, Heike Eschenbeck, Arnold Lohaus

**Affiliations:** 1 Department of Developmental Psychology, Bielefeld University, Bielefeld, Germany; 2 Department of Clinical Psychology and Psychotherapy, Medical School Berlin, Berlin, Germany; 3 Department of Educational Psychology and Health Psychology, University of Education Schwäbisch Gmünd, Schwäbisch Gmünd, Germany

**Keywords:** Gender Role Attitudes, Refugee Adolescents, Measurement Invariance, Well-Being

## Abstract

**Background:**

Assumed differences in gender role attitudes (GRAs) of German adolescents and refugee adolescents from the Middle East are often discussed, but rarely investigated. Presumed differences in GRAs across cultures and genders are assumed to be involved in emerging gender differences in well-being and mental health symptoms. Overall, appropriate measurements for investigating GRAs of adolescents with different cultural backgrounds are scarce.

**Methods:**

Hence, the present study exemplarily investigates (1) the measurement invariance (MI) of a German translation of the Social Role Questionnaire (SRQ) for German (*n* = 114) and German-speaking Middle Eastern refugee adolescents from Syria, Afghanistan, or Iraq (*n* = 115), using a Multiple Indicator Multiple Cause (MIMIC) model to account for age and gender. Moreover, (2) differences between GRAs of both groups, (3) relationships of GRAs with different facets of affective well-being, as well as (4) differences in these relationships between German and refugee adolescents are examined by extending the MIMIC-model to a full structural equation model (SEM).

**Results:**

Results indicate (1) that scalar MI for the SRQ can be assumed. Furthermore, (2) German adolescents show less traditional gender-linked GRAs than refugee adolescents, but no further differences in GRAs. Furthermore, no differences between the relationships of GRAs with well-being and mental health symptoms were found between the groups (4). Also, (3) GRAs showed no relation with any of the outcomes, but gender and age predicted mental health symptoms.

**Conclusion:**

The findings show that the SRQ is a useful measurement for investigating the GRAs of adolescents living in Germany and could be used in further cross-cultural research.

**Supplementary Information:**

The online version contains supplementary material available at 10.1186/s12888-023-05100-4.

## Background

With asylum applications reaching an all-time high since World War II, gender role attitudes of immigrants and refugees and the supposedly different attitudes between them and members of German society have been frequently discussed. Gender role attitudes (GRAs) are a person’s attitudes about the roles that each gender should take from a traditional perspective in terms of participation in society, the workplace, and in the home/family. Connected to these roles are beliefs about norms and appropriate behaviors for men and women (Baber and Tucker 2006). Possible differences in the attitudes between refugees and members of the German society are often seen as an obstacle to successful integration. However, most public debates on this topic ignore the lack of data as well as the heterogeneity of the groups and of the attitudes discussed. Moreover, gender role attitudes are considered important not only for the transition from one place to another, but also for the transition from childhood to adulthood experienced during puberty. In this developmental phase, GRAs play a role in adolescents’ social and academic progress (Fan and Marini 2000; Fuchs et al. 2021; Halimi et al. 2016). Furthermore, gender differences in facets of affective well-being, such as anger, sadness, anxiety, or in the form of positive affect (well-being) increase during puberty, and the increase is often considered to be associated with differences in the socialization of gender and associated roles (Fragoso and Kashubeck 2000; King et al. 2019).

Data on the GRAs of German adolescents and of refugee adolescents are scarce (Kretschmer 2018), which hinders reliable statements about possible differences in gender role attitudes and their associations with mental health in both groups. The insufficient data can be explained in part by the difficulty of measuring GRAs in adolescents from different cultural backgrounds, since gender roles are formed through socialization processes influenced by the respective cultural background. While there are some German panel studies that include questions measuring GRAs, their suitability for capturing the entire concept is questionable (Kretschmer 2018; Walter 2018; Weziak-Bialowolska 2015). Furthermore, to investigate the possible differences between cultures, the measurements must be carried out in an unbiased manner in all cultures studied so that conclusions can be drawn about their differences and similarities (Constantin and Voicu, 2015; Van de Vijver and Tanzer, 2004).

## Measuring gender role attitudes

The commonly used measurements of GRAs in Germany have shown two major drawbacks. First, they have been found to be outdated and not relevant to contemporary lifestyles (Walter 2018). In many of these more established measures (e.g., Skala zur Messung der normativen Geschlechtsrollen-Orientierung [Scale for measuring normative gender role orientation], GRO-Scale; (Krampen 1979), Attitudes towards Women Scale for Adolescents, AWSA; (Galambos et al. 1985), the traditional role model of the male breadwinner and the female caretaker was set as the standard, often reflected in the wording of the items (Baber and Tucker 2006; Walter 2018). While the traditional role models could be found in different cultures, including the German and the Syrian one, the adherence to these traditional models and the current way of life can differ profoundly within and between cultures and has changed over time (Alzoubi et al. 2019; Barth and Trübner 2018).

Second, they often do not capture the entire concept of GRAs in different populations unbiased (Kretschmer 2018; Walter 2018; Weziak-Bialowolska 2015). While there are some seemingly universal findings on gender role attitudes across cultures, e.g., that women and girls exhibit less traditional gender role attitudes than men and boys do (Baber and Tucker 2006, (Dotti Sani and Quaranta 2017), many of the available data on GRAs were collected in panel studies (e.g., World Value Survey [WVS], International Social Survey Program [ISSP]; for adolescents: Shell Youth Study, Children of Immigrants Longitudinal Survey in Four European Countries [CILS4EU]). These questionnaires contain only a small number of items, some of them focusing on the role of only one gender or certain areas of life. Because gender role attitudes affect several areas, such as engagement in work, society and politics, as well as the division of household tasks and care work for both genders on an individual and societal level, such a small number of items is rarely sufficient to capture all of these facets. This is even more questionable when considering that different cultures might consider different facets to be part of the respective gender roles (Han et al. 2019). As a result some of the previously mentioned measurement instruments have been shown not to measure gender role attitudes unbiased when used across cultures (Seddig and Lomazzi 2019). These biases occur among other things if a construct includes different aspects for different cultures or items were constructed to evoke an additional or ambiguous meaning (Boer et al. 2018).

Additionally, recent research suggests that these shortcomings of currently used measures are aggravated by the increasing complexity of gender role models which are due to developments such as the greater participation of women in the workforce and the more diverse family constellations in many societies, including the German or Syrian (Barth and Trübner 2018; Lokot 2018; Walter 2018). The shift away from traditional role models could lead to disagreement with the distribution of roles it contains, without necessarily disagreeing with other traditional attitudes, such as that women should be able to work and take care of children, but men are still better suited for leadership positions. This circumstance highlights the importance of covering attitudes towards more than the traditional breadwinner model or just one gender (Baber and Tucker 2006; Walter, 2018). Therefore, an appropriate measure should at least contain items that target behaviors and roles that are firmly associated with a particular gender (gender-linked), but target both genders.

### The social role questionnaire

The increasing complexity of gender roles and their different forms raises the question if they cannot be considered social roles which are not necessarily defined by a person’s gender. Many instruments do not allow this attitude to be expressed because they do not include items that reject the idea that gender is a meaningful category for assigning roles (gender-transcendent). Against this background, Baber and Tucker (2006) developed the Social Role Questionnaire (SRQ), which includes a scale to measure gender-transcendent attitudes independent of attitudes toward gender-linked norms and roles. Adding this scale to capture attitudes that reject gender as a decisive criterion for particular roles also provides an opportunity to free the measured attitudes from the notion that gender roles must be dichotomous. The rationale for this scale is based on the social constructivist approach, which states that gender is a concept construed through interactions of society, individuals, and their sex. It additionally highlights the importance of the environment for developing gender roles and GRAs. This impact of the environment is crucial in the concept of socialization, the process of internalizing the norms, rules, and behaviors from people in the environment. To reliably determine the prevailing attributes in different social environments (such as cultures) and to further investigate their relationships, it is important to have a measurement tool that is capable of measuring GRAs in different cultures without bias.

Recent studies have shown that the SRQ (Baber and Tucker, 2006) can be used with members of different cultures and at different ages, despite having originally been designed for use with young adults in the US (García-Sánchez et al. 2019; Naz 2021). Furthermore, the SRQ overcomes some of the listed shortcomings of older, but more established measures when it comes to the wording of the items and the domains they cover. It is comprised of two scales which cover the roles of both genders and allow to capture a more complex picture of gender role attitudes than many older instruments. At the same time, it is short enough to be used in larger studies covering a variety of concepts without taking up too much of the participants’ time. Despite these advantages, studies on its suitability for use in comparative research with adolescents of different cultural backgrounds (such as refugees living in Germany) have not yet been conducted.

### Gender role attitudes and relationships with affective well-being

As previously mentioned, socialization is not only seen as a reason for differences between cultures, but also as a crucial source for gender differences (Lengua and Stormshak 2000; Torsheim et al. 2006). Gender differences in different facets of affective well-being are often found by studies and emerge around the onset of puberty (﻿Ravens-Sieberer et al. 2009; Sweeting and West 2003; Torsheim et al. 2006). They are frequently explained by the socialization of certain behaviors adolescents learn as appropriate when forming their gender identities. Additionally, the roles associated with these gender identities might result in more experienced stress, as restrictive rules for girls could be an additional stressor, whereas behavioral norms for boys such as displaying more aggressive behaviors are assumed to aggravate negative outcomes of stressful situations (Baird et al. 2019; Fragoso and Kashubeck 2000; Jaehn et al. 2020). Therefore, gender role conformity could be related to the experience of affective well-being. Research and findings about these relationships are scarce. Jaehn et al. (2020) found that depressive symptoms, as well as symptoms of general anxiety disorder, are more prevalent in adult participants who held more traditional GRAs. King et al. (2021) found that men and women with egalitarian GRAs showed better mental health compared to those with more traditional attitudes. It is unclear if these relationships differ depending on the cultural background of adolescents.

### Aims of the present study

Based on this background the study focuses on three main questions: Can the SRQ be used in comparative research on attitudes towards gender roles in adolescents living in Germany? Is it furthermore legitimate to compare the groups (adolescents born in Germany and adolescent refugees from Middle Eastern countries) regarding their GRAs as measured with the SRQ? One prerequisite for investigations of any construct in samples with different cultural backgrounds is measurement invariance, as it is one requirement for unbiased cross-cultural analyses. In case of a non-invariant measurement, differences between the samples could either be due to differences between populations of different cultures or due to measurement bias and therefore would not allow correct conclusions about the actual differences between the populations (Meitinger et al. 2020) This is particularly important if the conclusions drawn from comparisons serve as a basis for further research or policy actions (Fuchs et al. 2021). Finally, are the GRAs of adolescents with different origins related to their affective well-being and do these relationships differ between cultural groups?

The following hypotheses emerge from these questions: First, because of previous success in cross-cultural studies (Naz et. al. 2021) and its social constructivist nature the SRQ is expected to be measurement invariant between German natives and refugee adolescents from Middle Eastern countries living in Germany (Hypothesis 1). Second, based on previous findings (Kretschmer 2018) refugee adolescents will show more traditional GRAs then their German peers if a comparison is permissible (Hypothesis 2). Third, gender role attitudes are related to affective well-being in both groups, with less traditional roles being associated with greater well-being (Hypothesis 3). Fourth, continuing the line of reasoning, there are differences in the relationships of GRAs and affective well-being between both groups (Hypothesis 4).

## Method

### Sample and procedure

#### Refugee subsample

Participants of the refugee subsample were collected as part of a larger project on the health-related development of refugees in Germany. The overall project is a panel study with three data collection waves. During the second wave new participants were included to account for the assumed high attrition rate during the COVID-19 pandemic. As the focus of the current study is on a cross-sectional measurement invariance analysis the subsamples consist of a combination on all first-time participants similar to a repeated cross-sectional design. During the first wave of data collection (January to October 2019), participants filled out computerized questionnaires in group settings either at schools or in housing facilities. Active informed consent from the parents and participants was obtained. This process might have led to a smaller sample size (Pokorny et al. 2001), but was in line with the requirements of the ethics commission. Participants chose a language to complete the SRQ and other questionnaires (German, Arabic, Persian, or Kurmancî/Sorani Kurdish). If needed, trained research assistants fluent in the participants’ native language could offer help. The second wave of data collection began in early 2020 and was conducted in a similar way. With the beginning of the COVID-19 pandemic, the data collection procedure was adapted to the new circumstances not allowing for the previously used group setting. Participants received the printed questionnaires via mail and were supported via telephone during assessment to minimize interpersonal contact. Support via telephone was again given by trained research assistants fluent in the respective language of the participants. Adolescents received a €20 voucher as incentive for their participation.

First-time participants from both waves were included in this study. This resulted in a total sample of *N* = 308 adolescents. Of these, *n* = 60 participants chose to use one of the various language versions other than German (Arabic, *n* = 42; Persian, *n* = 10; Kurmancî Kurdish, *n* = 8). Because none of the subgroups using other languages reached a sufficient sample size to be part of a test for measurement invariance, they were excluded from the following analyses and measurement invariance was therefore only tested for the German translation. Thus, the sample consisted of *N* = 248 participants. The possible effects this selection might have on the sample and results of this study will be discussed later. Regarding missing values, participants who lacked more than 20% in each scale or more than one item missing on the remaining scales of well-being or the mental health symptoms (*n* = 124) or had not indicated their age (*n* = 9) or gender (*n* = 2) were excluded. This resulted in a final sample of *N* = 113 participants. The high exclusion rate is due to the strict criteria for missing values, which was chosen to minimize missing values in the measurement invariance analysis. No significant differences in age or gender between the final sample and the excluded participants were found. The final sample consisted of 52% boys (48% girls, 0% divers), and the mean age was *M* = 14.32 years (*SD* = 1.88). Participants came from Syria (*n* = 51), Iraq (*n* = 34), Afghanistan (*n* = 9), or did not indicate any country of origin but were included based on the recruitment process only addressing those from Middle Eastern countries (*n* = 19).

### German subsample

Participants for the German sub-sample were recruited in schools in 2019 and 2020. During 2019, data collection was similar to the one described for participants with a refugee background in school settings with *n* = 89 participants receiving the German version of the questionnaires of interest. During 2020, data collection was conducted via the online tool Qualtrics with a shortened questionnaire including only the SRQ, the Revised German Stress and Coping Questionnaire for Children and Adolescents (SSKJ; (Lohaus et al. 2018), and demographic information (*n* = 42). After combining both samples, the same exclusion criteria for missing data (> 20% on SRQ scales or more than one item missing on the remaining scales, *n* = 4) were applied. Participants not born in Germany (*n* = 4) or whose parents were both not born in Germany were excluded (*n* = 10), resulting in a final German sample of *N* = 113 adolescents. The exclusions were made to secure that the German sample was highly likely to contain only participants whose mother language was German. Participants in the German sample consisted of 58% boys (42% girls, 0% diverse) with a mean age of *M* = 13.92 years (*SD* = 1.54). Of these*, n* = 11 had one parent not born in Germany. Again, no significant differences in gender and age between the final sample and those excluded from the analyses were found.

Ethics approval from the ethics committees of the respective universities was granted for all data collection procedures. All participants’ parents had given informed consent for their children to participate in the study.

### Measurements

Since the data were collected as part of a large-scale study, only those questionnaires relevant for the present study will be presented in further detail. All participants were asked to indicate their gender (boy, girl, or diverse) and age. German participants indicated if they themselves and their parents were born in Germany (see Additional File 1 in the supplementary materials for an overview of sociodemographic questions). For the refugee sample, computerized measurements were provided in German, Arabic, Persian, Kurmancî Kurdish, and Sorani Kurdish; paper–pencil questionnaires were provided in German and Arabic only.

### Gender role attitudes

The Social Role Questionnaire (SRQ; Baber and Tucker 2006) measures participants’ gender-linked and gender-transcendent attitudes. Participants state their agreement with statements such as “Mothers should work only if necessary” (gender-linked) or “We should stop thinking about whether people are male or female and focus on other characteristics (e.g., kindness, ability, etc.)” (gender-transcendent; reverse coded) on an eleven-point Likert scale ranging from *not at all* (0%) to *completely agree *(100%). The German translation of all items was conducted following a back-translation procedure described in Hambleton (2001) and can be found attached (Additional File 1, for the original items see Barber and Tucker 2006). After re-coding of the five reverse coded gender-transcendent items, a lower value on both scales represents a less traditional attitude toward gender roles. Table 1 shows the results for Cronbach’s α for each scale and subsample of the current study. Overall, reliability was acceptable, but slightly better in the German subsample (Table 1). In general, the reliabilities of the scales are slightly higher than those of the original sample in which the gender-linked (Cronbach’s α = 0.77) and gender-transcendent scale (Cronbach’s α = 0.65) accounted for 41% of variance (Baber and Tucker 2006). Subsequent studies with samples from different populations by Naz et al. (2021) and Lopez-Cepero et al. (2013) found slightly higher internal consistencies, ranging from 0.70–0.83 for the gender-transcendent and from 0.77–0.96 for the gender-linked scale.

**Table 1:** Means, SDs, and Reliabilities (Cronbach’s α) of used measurements by group

	Refugee Sample		German Sample	
	*M(SD)*	α	*M(SD)*	α
Gender-transcendent	15.58 (10.84)	.76	24.61 (17.85)	.91
Gender-linked	49.98 (17.07)	.82	31.58 (17.46)	.86
Anger	5.99 (2.00)	.75	7.61 (2.55)	.81
Sadness	6.66 (2.10)	.63	6.98 (2.50)	.79
Anxiety	7.24 (2.07)	.58	7.81 (2.22)	.66
Well-Being	9.82 (1.95)	.62	11.16 (1.55)	.74

Cronbach’s α was considered acceptable at .50 -.60 based on the small number of items per scale (Barber and Tucker 2006)

### Affective well-being

Facets of affective well-being were measured with the Revised German Stress and Coping Questionnaire for Children and Adolescents (Lohaus et al. 2018). For this study, only the symptom scales measuring well-being (e. g., “How often have you been happy in the last week?”), anxiety (e.g., “How often have you been nervous in the last week?”), sadness (e.g., “How often have you felt sad in the last week?”), and anger (e.g., “How often have you been angry in the last week?”) were part of the analyses. Each scale contains four items asking about the occurrence of certain states and feelings during the last week with answer options ranging from *never* (1) to *several times* (3). Reliability for the symptom scales were partly low to acceptable and again slightly better in the German sample (Table 1). Different language versions of the SSKJ have been used in cross-cultural research and proved useful to measure affective well-being in different cultures (Gillé et al. 2021).

### Analyses

As described above, cases with > 20% missing values were excluded. The remaining missing values (3%) only occurred in the refugee sample, mainly on the scales of the SRQ (90% of the remaining missing values). This might be because the longer gender-linked scale allowed for more than one missing value as an exclusion criterion, the SRQ’s item formulation was more complex than in the SSKJ scales, and the SRQ was taken later in the data collection. The affective well-being scales accounted for the remaining 10% of missing values. All missing values were handled by using full maximum likelihood (FIML) estimation.

To examine the level of measurement invariance (MI; Hypothesis 1), a MIMIC-model including gender and age as covariates was calculated. This approach is similar to multi-group Confirmatory Factor Analysis (CFA) but allows for the inclusion of covariates. Because MIMIC-models have been shown to be biased in the absence of metric invariance (Kim et al. 2012), simple multi-group CFA-models were used to safeguard the analyses by testing for configural, metric, and scalar measurement invariances without covariates.

As metric measurement invariance was given, results of the following MIMIC-model are considered trustworthy. In line with the multi-group CFA approach of testing MI, a model with increasing numbers of constraints was estimated for each level of potential MI. At the first level (configural MI), no equality constraints were imposed on any parameter, and a good fit of CFI > 0.95, RMSEA < 0.05, and SRMR < 0.08 (Hu and Bentler 1999) indicates an equal structure of the model for both groups. To test metric MI, factor loadings are set equal for both groups and a chi-square difference test is calculated to compare the more constrained model with the less constrained model. A significant change in chi-square would result in a rejection of the tested level of measurement invariance. Additional cutoffs for differences in alternative fit indices such as CFI (cut-off difference:Δ = -0.01), RMSEA (cut-off difference: Δ = 0.015), and SRMR (cut-off difference: Δ= 0.030) can be used to inform the decision about rejecting or accepting measurement invariance (Putnick and Bornstein 2016). A consistently good model fit, as indicated by the test, implies metric MI and allows for comparisons of structural relationships between both groups. Additionally, to test scalar MI, intercepts of the model are set equal for both groups and the model is tested against the less restricted model of metric MI. Again, if the model fit is consistently good, as tested with the chi-square difference test, the scalar level of MI is reached. This allows comparison of means between groups.

The grouping variable was the group membership (refugee sample vs. German sample). In addition, results of previous studies suggested differences between male and female participants (Baber and Tucker 2006). Because the sample size was not sufficient to form four groups, gender was included as a dummy-coded covariate with female participants as the reference group. Given trends toward less traditional gender role attitudes among older adolescents and age-related changes of affective well-being, age was included as an additional covariate (Crouter et al. 2007; Ravens-Sieberer et al. 2009). To avoid choosing an incorrect reference item, effect coding was used with refugees as the reference group. This approach, described by Little et al. (2006), allowed for scaling of the latent variable without the disadvantage of having to choose a reference item whose invariance status is unknown. The approach also allows for easy comparison of potential differences in the latent means of both groups if scalar MI is given. After establishing the level of measurement invariance, the MIMIC-model was extended by including well-being, sadness, anxiety, and anger as manifest outcomes to the model. In this way, the hypothesized relationships between the two factors—gender-linked and gender-transcendent GRAs with affective well-being—were tested for both groups (Hypothesis 3). Again, gender and age served as covariates. Finally, possible differences in the relationships between GRAs, gender, and age with the different facets of affective well-being between adolescent refugees and German adolescents were tested by constraining the paths to be equal in both groups (Hypothesis 4). A deterioration in model fit, again tested by chi-square difference test, implies different relationships between the variables for each group.

All models were computed using lavaan 0.6–9 (Rosseel 2012) in R Studio. Non-normal distribution of variables was addressed by using a robust Maximum Likelihood estimator (MLR).

## Results

First, measurement invariance was investigated by using MIMIC-models for both groups (configural level) and by further adding constraints (metric and scalar level).

The fit of all MIMIC-models was acceptable to good, χ^2^ (149) = 204.44, CFI = 0.95, RMSEA = 0.06, and—supporting Hypothesis 1—scalar measurement invariance was given, Δχ^2^ (10) = 10.03, *p* = 0.438. The model comparison showed no significant differences between the less restricted models for lower levels of measurement invariance and the final model (Table 2).

**Table 2:** Model fit and results of model comparisons to establish measurement invariance

	Model-fit				Model comparison					
Model	χ^2^ (*df*)	CFI	RMSEA (90% CI)	SRMR	Model comp	Δχ^2^ (Δ*df*)	ΔCFI	*p*	ΔRMSEA	ΔSRMR
M1: Configural Invariance	180.28 (128)	.95	.06 (.04-.08)	.06	-	-	-	-	-	-
M2: Metric Invariance	193.93 (139)	.95	.06 (.04-.08)	.07	M1 vs. M2	13.17 (11)	.00	.282	.00	-.01
M3: Scalar Invariance	204.44 (149)	.95	.06 (.04- .08)	.07	M2 vs. M3	10.03 (10)	.00	.438	.00	.00

Because measurement invariance reached the scalar level, a comparison between the means of both groups to test Hypothesis 2 was permissible. The effect coding allowed for a test by looking directly at the means of each latent variable in both groups. The refugee group scored significantly higher on the gender-linked scale (*M* = 6.29,* SD* = 1.82) with its latent mean being 2.75 units higher than this of the German sample (*M* = 3.30, *SD* = 2.02), *p* = 0.004. The lower values of the refugee group on the gender-transcendent scale (*M* = 2.34, *SD* = 1.81) did not differ significantly from those of the German group (*M* = 3.34, *SD* = 3.11), *p* = 0.704.

**Fig. 1:**
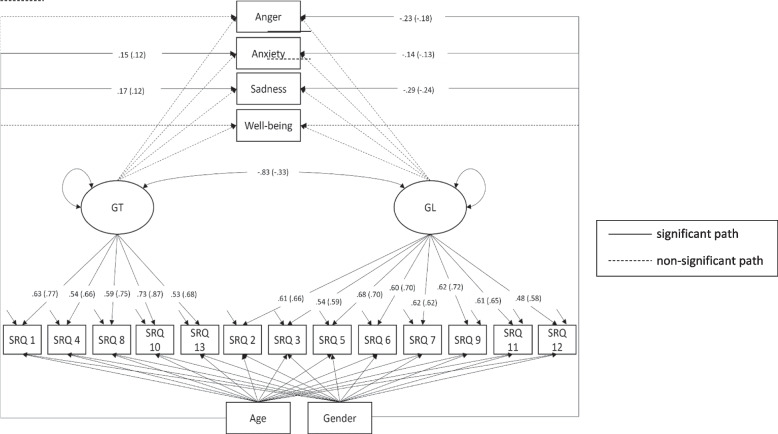
Final SEM. Note. For reasons of simplification non-significant coefficients are not displayed. All displayed coefficients are standardized and *p* < .05; brackets display coefficients of the German sample. Gender coded 0 for girls and 1 for boys. GT = gender-transcendent factor. GL = gender-linked factor. SRQ = Social Role Questionnaire item and its respective number. χ^2^ (249) = 310.85, CFI = .95, RMSEA = .05

The scalar model was extended to a structural equation model (SEM) to test the relationships between GRAs and affective well-being in both groups. The added paths were allowed to vary freely between the adolescent refugee group and the German adolescents group to test Hypothesis 3. The model showed an acceptable to good model fit, χ^2^ (233) = 296.90, CFI = 0.95, RMSEA = 0.05. In a second step, the paths of the model were restricted to be equal for both groups to investigate if the paths differ significantly between the groups (Hypothesis 4). This model fit the data acceptably well, χ^2^ (249) = 310.85, CFI = 0.95, RMSEA = 0.05, and a comparison with the less constrained model did not show a significantly worse fit, Δχ^2^ (16) = 13.85, *p* = 0.610. Hence, no moderation by group membership on the relationships between age, gender, gender role attitudes, well-being, and mental health symptoms was found. Figure 1 shows the estimates for all significant paths in the final model.

Age and gender significantly predicted sadness and anxiety, whereas gender alone predicted anger (Table 3). Girls reported a greater amount of mental health symptoms. Similarly, older age was related to reporting more mental health symptoms.

**Table 3:** Path Coefficients of the full scalar invariance model with paths being restricted to be equal between groups

Dependent Variables	Independent Variables	*B* (*SE*)	β	
			Refugee	German
Well-Being				
	Gender-transcendent GRAs	0.09 (0.04)	.08	.17
	Gender-linked GRAs	-0.05 (0.07)	-.05	-.06
	Gender	0.03 (0.23)	.01	.01
	Age	-0.08 (0.07)	-.07	-.08
Sadness				
	Gender-transcendent GRAs	-0.12 (0.07)	-.10	-.15
	Gender-linked GRAs	-0.09 (0.10)	-.08	-.07
	Gender	-1.22 (0.29)*	-.19	-.24
	Age	0.19 (0.09)*	.17	.12
Anxiety				
	Gender-transcendent GRAs	-0.07 (0.08)	-.06	-.09
	Gender-linked GRAs	-0.17 (0.11)	-.15	-.15
	Gender	-0.58 (0.28)*	-.14	-.13
	Age	0.18 (0.08)*	.15	.12
Anger				
	Gender-transcendent GRAs	-0.08 (0.09)	-.08	-.10
	Gender-linked GRAs	-0.16 (0.11)	-.15	-.12
	Gender	-0.93 (0.29)*	-.23	-.18
	Age	0.15 (0.08)	.14	.09

^*^ *p* < .05. GRAs = Gender role Attitudes

## Discussion

The study established the measurement invariance of a translated version of the Social Role Questionnaire to prove its usefulness as a measurement for gender-role attitudes of German adolescents and German-speaking adolescent refugees from Syria, Afghanistan, or Iraq (1). As scalar measurement invariance for both samples was achieved, a comparison was made between GRAs of adolescent refugees and German adolescents (2). Results show that only gender-linked attitudes were significantly higher for the adolescent refugees, and no significant differences in gender-transcendent GRAs between both groups were found, which only partly supports Hypothesis 2. Subsequently, the relationships of GRAs and the affective well-being was investigated (3). While neither GRAs predicted any facet of affective well-being, gender and age were found to predict some of them. In a final step, these relationships were examined regarding possible differences between the relationships of both groups (4). Such differences were not found.

### Measuring gender role attitudes in adolescents with refugee experience and their german peers

To answer questions about differences and similarities in GRAs of adolescents with different cultural backgrounds, such as refugees from Middle Eastern countries and German natives, it was necessary to examine the usefulness of the SRQ (Baber and Tucker 2006) by demonstrating its measurement invariance. In line with Hypothesis 1, the results show that scalar measurement invariance can be assumed, which makes statements about differences and relationships possible. Therefore, group-differences in the latent means of the gender-linked and gender-transcendent scales were examined.

No significant differences in gender-transcendent attitudes between the two groups were found, but refugee adolescents reported more traditional gender-linked attitudes than their German peers. In terms of gender-transcendent attitudes, adolescents—regardless of their background—agreed on the extent of importance that should be attached to gender as a category for distinguishing social roles. Thus, the results only partly confirmed Hypothesis 2. Naz et al. (2021) found the same pattern of differences between British and Pakistani young adults. They link their findings to a more traditional upbringing of the Pakistani participants based on the values and role expectations of their culture of origin in case of gender-linked attitudes. However, they also indicated similar acceptance of gender egalitarianism, as measured with the gender-transcendent scale, which Naz et al. (2021) attributed to the contact with a more individualistic culture. These findings can be related to the findings of Arends-Tóth et al. (2009), who found that attitudes toward gender norms and behaviors show varying degrees of agreement depending on the domains they address, such as marriage, family or employment, which are addressed in the scales of the SRQ (Baber and Tucker 2006). Gender role attitudes are often passed on from parents and their more traditional attitudes prevail to some extent (Kretschmer 2018). However, they are not static but dynamic and change depending on the situation and over the lifespan (Arends-Tóth et al., 2009; Fan and Marini 2000). Even though the adolescent refugees show more traditional gender-linked attitudes, it can be argued that both groups of adolescents are similarly open to disregarding gender as a useful category, which allows for a certain flexibility. Studies of GRAs of immigrants living in Germany (Idema and Phalet 2007; Kretschmer 2018), see also Phinney and Flores (2002) for similar results in Hispanic immigrants in the US, found an association between less traditional GRAs and integration into the host society. A common proxy- measure of integration is use of language (Doucerain et al. 2016). As the current study relied solely on refugee adolescents who felt comfortable enough to fill out the SRQ in German, the sample analyzed in this study could be well integrated. This could reflect in their similar attitudes towards gender-transcendent gender-roles. Cross-cultural exchange might therefore foster less traditional attitudes in adolescent refugees in Germany. It is, however, not possible to rule out a possible influence of social desirability on the answers given to the SRQ. Even though Baber and Tucker (2006) found no association between the SRQ and a social desirability scale they tested a very different sample. Further studies should therefore investigate the effect of social desirability, as well as the effect and direction of the relationship between integration and less traditional gender role attitudes based on longitudinal studies.

Hypotheses 3 and 4 explored possible implications of GRAs for the life of adolescents by examining associations with facets of affective well-being, such as well-being, sadness, anxiety, and anger. Although previous studies (Baird et al. 2019; Fragoso and Kashubeck 2000; Jaehn et al. 2020; King et al. 2019; Lengua and Stormshak 2000) suggested that gender differences in these outcomes may be due to differences in GRAs, the present results could not confirm this assumption. None of the outcome variables were predicted by gender-linked or gender-transcendent attitudes. To explain the lack of associations between GRAs and affective well-being, a look at studies with adult populations might be helpful.

Various of those studies (e.g., Soltanpanah et al. 2018; Sweeting et al. 2014; van de Vijver 2007) show that it is not the GRAs themselves, but a possible discrepancy between the gender role lived and the GRAs represented, as well as a discrepancy between one’s own GRAs and the GRAs of the environment, which influence the well-being or mental health of men and women. These studies suggest that the match/mismatch with the general GRAs of the environment or the lived gender role are most relevant. Further studies should include measurements of such discrepancies to investigate a possible impact. Additionally, refugees themselves do not consider the differences in GRAs between themselves and German natives to be significant for their everyday lives (Forschungsbereich beim Sachverständigenrat deutscher Stiftungen für Integration und Migration [SVR-Forschungsbereich], 2019). This could further weaken the link between the GRAs and affective well-being and might offer an additional explanation for the absence of a moderation by group membership, as adolescent refugees might also not consider differences in GRAs to be important. Additionally, the internal consistencies of the affective well-being scales were partly low, restricting the meaningfulness of the findings. Nonetheless, with regard to gender differences in affective well-being, the results show similar findings to previous studies (Ravens-Sieberer et al. 2009; Torsheim et al. 2006). Girls reported more sadness, anxiety, or anger than boys. Anxiety and sadness were also positively predicted by age, which is also in line with findings by Ravens-Sieberer et al. (2009). Surprisingly, when looking at well-being, no gender difference was found. Although girls seem to face more negative mental health outcomes, their well-being was equal to that of boys. Further research is needed to understand gender differences in affective well-being emerging during puberty and their possible relationships with GRAs. In (cross-cultural) research, further insights might be gained by including additional background variables such as socioeconomic background, religious affiliation, or GRAs of the immediate and broader environment (Kågesten et al. 2016).

### Strengths and limitations

The investigation of measurement invariance of the German translation of the SRQ as a prerequisite for cross-cultural comparisons is a first important step to gain evidence-based insights into the differences and similarities of GRAs of adolescents living in Germany. Nonetheless, further differentiation of the samples, e.g., into sub-groups by gender, different countries of origin or ethnicities would have further improved the present study.

Due to the small number of participants, a further diversification was not possible but should be a priority in upcoming studies as the population of refugee adolescents in Germany is very heterogenous. This is especially true when considering that the final sample of refugee adolescents consisted of only those who felt comfortable to fill out the questionnaire in German. Their language skills might reflect a longer stay in Germany, arriving at a younger age or a general ability or will to orient toward the German culture. They therefore might be more similar to the German sample as expected in their understanding of the SRQ and GRA. The relatively small sample size was deemed appropriate for the present analyses by rule of thumb, but a larger sample would additionally allow further investigations of more complex relationships of gender role attitudes of adolescents in Germany, e.g., with gender, cultural background, country of origin, or interactions of these variables. Nonetheless, since studies of measurement invariance are more concerned with fitting the overall model and less with individual parameters, the power was still considered appropriate, and the sample of adolescent refugees is a unique feature of the study. Furthermore, the small amount of completed SRQs in Arabic, Farsi or any of the Kurdish languages made it impossible to test the MI of these translations from the German version. Here the linguistic differences are assumed to add to the measurement variance and testing MI would therefore be of great importance (Boer et al. 2018). So far, no comparisons between the results of these versions can be made.

Because the German translation of the SRQ used in this study was administered to an overall younger sample than it was constructed for, the investigation provided further insight into construct validity in addition to its reliability. The good model fit can be taken as a strong sign of construct validity (good model fit, acceptable values of internal consistency), but there is still little information on other forms of validity. Nonetheless, the findings of the current study suggest that the German version of the SRQ is a reliable and valid measurement for different groups of adolescents with different cultural backgrounds. It is therefore helpful in investigating GRAs in general (e.g., possible differences between those fleeing from other countries to Germany and its native population) and associations with other variables such as religious beliefs or career choices. Furthermore, cross-cultural research could also use the SRQ to examine whether and how GRAs change over time and if they converge across these groups during their development within the same country.

## Conclusion

Measuring gender role attitudes of adolescents from different cultural backgrounds has been difficult so far, due to a lack of suitable questionnaires. The present study found that the SRQ (Baber and Tucker, 2006) is a suitable measure with the present sample of German and German-speaking refugee adolescents from Syria, Afghanistan, or Iraq. Since scalar measurement invariance could be demonstrated, gender role attitudes of German adolescents could be compared to those to adolescents with refugee experience. Findings suggest that adolescents with refugee experience only had more traditional gender-related attitudes, while both groups had equal scores on attitudes toward gender as a defining category of social roles. Similarly, there were no differences in the relationships between gender role attitudes, gender, age, and affective well-being in the two groups. Among adolescents living in Germany, including those who arrived as refugees, gender and age were related to single facets of affective well-being, but GRAs were not.

### Supplementary Information

**Additional file 1. **Overview of sociodemographic questions. Sociodemographic questions of the German subsample.

## Data Availability

The dataset generated and analyzed during the current study is not publicly available due the fact that it constitutes an excerpt of research in progress of the YOURGROWTH project. All datasets generated for the YOURGROWTH project will be made available at the end of said project on the RCD at ZPID (https://rdc-psychology.org/). The datasets generated during and/or analyzed during the current study are available from the corresponding author on reasonable request. For any information on the current status of the dataset please contact the corresponding author.

## Abbreviations

AWSA: Attitudes towards Women Scale for Adolescents
CFA: Confirmatory Factor Analysis
CFI: Comparative Fit Index
CILS4EU: Children of Immigrants Longitudinal Survey in Four European Countries
COVID-19: Coronavirus Disease 2019
FIML: Full Information Maximum Likelihood
GRA: Gender Role Attitudes
GRO-Scale: Scale for measuring normative gender role orientation
ISSP: International Social Survey Program
MI: Measurement Invariance
MIMIC: Multiple Cause Multiple Indicators
MLR: Robust Maximum Likelihood
RMSEA: Root Mean Square Error of Approximation
SEM: Structural Equation Model
SRMR: Standardized Root Mean Square Residual
SRQ: Social Role Questionnaire
SSKJ: Revised German Stress and Coping Questionnaire for Children and Adolescents
SVR-Forschungsbereich: Advisory Council-Research Department (Forschungsbereich beim Sachverständigenrat deutscher Stiftungen für Integration und Migration)
WVS: World Value Survey
