# Supplementary material for: Gender role attitudes and well-being of German and refugee adolescents—same or different?
Source: BMC Psychiatry. 2023 Sep 8;23:663. doi: 10.1186/s12888-023-05100-4 (PMC10492273; doi:10.1186/s12888-023-05100-4)
Supplement: Supplementary file 1 — Additional file 1. Overview of sociodemographic questions. Sociodemographic questions of the German subsample. [file 12888_2023_5100_MOESM1_ESM.docx]

ESM German Translation of the Social Role Questionnaire

| German translation | Item No. | Scale |
| --- | --- | --- |
| Menschen können unabhängig vom Geschlecht sowohl aggressiv als auch fürsorglich sein. | 8 | GT |
| Menschen sollten unabhängig von ihrem Geschlecht gleichbehandelt werden. | 10 | GT |
| Die Freiheit, die Kindern gegeben wird, sollte durch ihr Alter und ihre Reife nicht durch ihr Geschlecht bestimmt werden. | 1 | GT |
| Aufgaben rund um das Haus sollten nicht nach Geschlecht zugeteilt werden. | 4 | GT |
| Wir sollten aufhören darüber nachzudenken, ob Menschen männlich oder weiblich sind und auf andere Eigenschaften fokussieren. | 13 | GT |
| Die Hauptverantwortung eines Vaters ist es, seine Kinder finanziell zu unterstützen. | 3 | GL |
| Männer sind sexueller als Frauen. | 7 | GL |
| Manche Arten von Arbeit sind einfach nicht für Frauen geeignet. | 2 | GL |
| Mütter sollten die meisten Entscheidungen darüber treffen wie Kinder erzogen werden. | 6 |  |
| Mütter sollten nur arbeiten, wenn es nötig ist. | 12 | GL |
| Mädchen sollten mehr beschützt und bewacht werden als Jungen. | 11 | GL |
| Nur manche Arten von Arbeit sind sowohl für Männer als auch Frauen geeignet. | 5 | GL |
| Für viele wichtige Berufe ist es besser, Männer statt Frauen auszuwählen. | 9 | GL |

Note. GL = gender-linked. GT = Gender-transcendent. Item No. refers to the position of the item in the questionnaire.
